# *ALK*-Negative Anaplastic Large Cell Lymphoma Presenting as Disseminated Intravascular Coagulation and Hemophagocytic Lymphohistiocytosis: A Potentially Fatal Presentation

**DOI:** 10.1155/2018/3465351

**Published:** 2018-04-11

**Authors:** Uroosa Ibrahim, Amina Saqib, Maryam Rehan, Jean Paul Atallah

**Affiliations:** ^1^Department of Hematology and Oncology, Staten Island University Hospital, 475 Seaview Avenue, Staten Island, New York, NY 10305, USA; ^2^Department of Pulmonary and Critical Care, Staten Island University Hospital, 475 Seaview Avenue, Staten Island, New York, NY 10305, USA; ^3^Department of Medicine, Staten Island University Hospital, 475 Seaview Avenue, Staten Island, New York, NY 10305, USA

## Abstract

Hemophagocytic lymphohistiocytosis (HLH) is a life-threatening disorder that can be familial in etiology or a result of infections, malignancy, and autoimmune or inflammatory disorders. Disseminated intravascular coagulation (DIC) is common in patients admitted to intensive care units and can confound and delay the diagnosis of HLH. We present a case of a 69-year-old female who presented with dyspnea and malaise. Her condition declined rapidly with laboratory parameters consistent with DIC. In addition, she had a ferritin of 32,522 ng/mL, low haptoglobin, and elevated LDH, and bone marrow biopsy showed hemophagocytic lymphohistiocytes. She was started on HLH-directed therapy, and later, a diagnosis of *ALK*-negative anaplastic large cell lymphoma was made on an excisional inguinal lymph node biopsy specimen. Our case emphasizes the importance of prompt recognition, diagnosis, and treatment of HLH while workup for a primary disorder is still being pursued.

## 1. Introduction

Disseminated intravascular coagulation (DIC) is common in critically ill patients and is usually secondary to sepsis. It can potentially confound a diagnosis of a fatal and uncommon disorder such as hemophagocytic lymphohistiocytosis (HLH). Both entities are clinicopathologic diagnoses with shared coagulation system abnormalities. Mortality from multiorgan failure can occur in up to seventy percent of patients [1]. We present a case of DIC initially managed supportively as being secondary to sepsis, with workup leaving HLH secondary to anaplastic large cell lymphoma (ALCL).

## 2. Case

A 69-year-old female with no known past medical history was brought to the hospital by ambulance for worsening shortness of breath and lethargy. At presentation, she was tachycardic, hypotensive, and hypoxic. Laboratory workup showed hemoglobin 13 g/dL, white blood cell count 2.61 × 10^9^/L, platelets 92 × 10^9^/L, alkaline phosphatase 212 IU/L, aspartate aminotransferase 166 IU/L, alanine aminotransferase 61 IU/L, and a normal coagulation profile.

**Figure 1:**
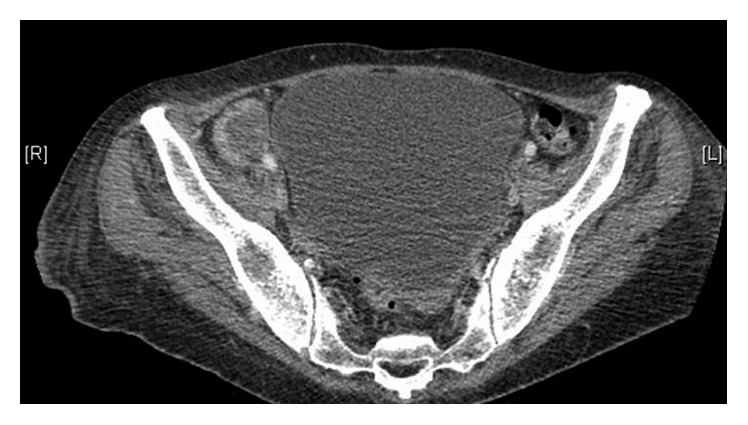
Right inguinal adenopathy.

**Figure 2:**
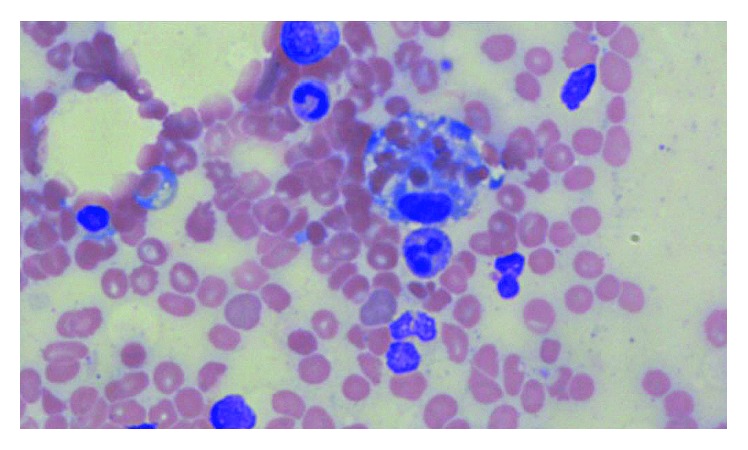
Hemophagocytosis on bone marrow biopsy.

Over the ensuing week, the patient's pancytopenia worsened (Table 1). PTT was up to 99 seconds, PT 13.7 seconds, and fibrinogen 84 mg/dL. A computed tomography (CT) scan of the abdomen showed a right superficial inguinal centrally necrotic nodal mass measuring 5.3 × 2.3 cm and a right deep inguinal lymph node measuring 3.0 × 2.3 cm (Figure 1). Several incompletely characterized splenic masses were also seen. A core needle biopsy of the lymph node showed necrotic soft tissue with focal granulomatous reaction. Infectious disease workup was negative. Further studies showed a ferritin level of 32,522 ng/mL, a haptoglobin level of <20 mg/dL, a lactate dehydrogenase level of 982 IU/L, and low natural killer cell activity. A bone marrow biopsy showed many histiocytes and significant hemophagocytic macrophages (Figure 2). A diagnosis of concomitant disseminated intravascular coagulation and hemophagocytic lymphohistiocytosis was made. Dexamethasone 10 mg/m^2^ was started, and she received two doses of etoposide 150 mg/m^2^, resulting in a decrease in ferritin to 19,947 ng/mL and normalization of coagulation parameters.

**Figure 3:**
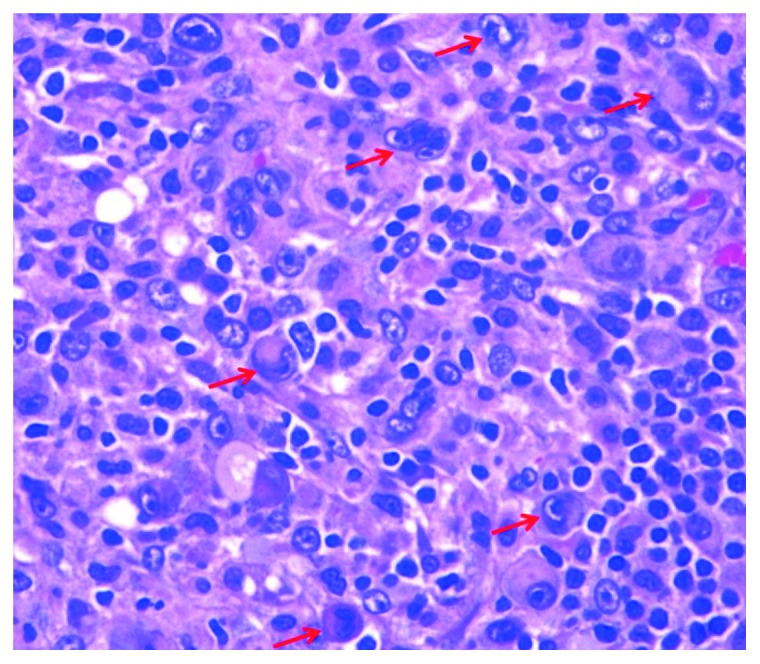
Right inguinal lymph node biopsy showing large pleomorphic and anaplastic cells.

An excisional inguinal lymph node biopsy showed an atypical lymphoid infiltrate with extensive geographic necrosis. Large pleomorphic and anaplastic cells with irregular nuclei and finely dispersed chromatin were identified (Figure 3). The atypical large cells were positive for CD30, CD2, CD4, CD43, MUM-1, and CD45 (variable). There was no significant expression of CD3, CD5, CD7, CD8, or EMA and *ALK-1*, CD20, PAX-5, CD79a, CD10, BCL-6, CD138, CD15, or BCL-2. The findings were consistent with *ALK*-negative anaplastic large cell lymphoma.

The treatment of choice for HLH is treating the underlying cause. The patient initially declined chemotherapy given the rapidly declining functional status. However, after three weeks of rehabilitation, her functional status improved significantly, and thereafter, she received chemotherapy treatment with the CHOP (cyclophosphamide, doxorubicin, vincristine, and prednisone) regimen. A positron emission tomography (PET) scan after four cycles showed resolution of the right inguinal lymphadenopathy and the splenic lesions.

## 3. Discussion

Primary systemic ALCL is a type of peripheral T-cell lymphoma which comprises about 2 percent of all non-Hodgkin's lymphomas. These are generally aggressive and four clinical subtypes exist: anaplastic lymphoma kinase- (*ALK*-) positive ALCL, *ALK*-negative ALCL, breast implant-associated ALCL, and primary cutaneous ALCL [2]. Cases involving the *ALK* gene translocation located on chromosome 2p23 fare significantly better compared to *ALK*-negative cases. Several translocations may be involved, the most common being t(2;5), that causes the fusion of the nucleophosmin (*NPM*) gene (5q35), coding for a nucleolar phosphoprotein, with the portion *ALK* on chromosome 2p23 that encodes the *ALK* cytoplasmic domain. The *NPM-ALK* fusion product acts as a constitutively active tyrosine kinase. In contrast, the *ALK*-negative ALCL patients may have the t(6;7)(p25.3;q32.3) translocation involving the *DUSP22* gene and the *FRA7H* fragile site in up to 30 percent of cases [3].

Given the aggressive nature of the disease, ALCL patients present with rapidly progressive adenopathy and systemic symptoms of fever, weight loss, and night sweats. Compared to B-cell NHLs and HLs, T-cell NHLs are reported to be complicated with HLH during their course of the disease, and this confers a worse prognosis. ALCL is seen most often in females and is associated with advanced stage at presentation, frequent B symptoms, organomegaly and lymphadenopathy, cytopenias, liver dysfunction, coagulopathy, higher proportion of bone marrow infiltration, and reduced overall survival with or without definitive chemotherapy [4].

Hemophagocytic lymphohistiocytosis results from uncontrolled activation of cytotoxic T cells resulting in a cytokine storm. It can be familial or secondary to an underlying disorder and has a mortality rate of up to 70% from multiorgan failure. Clinical and laboratory features include fever, splenomegaly, cytopenias, hypertriglyceridemia, hypofibrinogenemia, hemophagocytosis, low or absent NK-cell activity, hyperferritinemia, and an elevated soluble CD 25. Lymphoma is known to trigger HLH [5]. In the latter case, HLH- and lymphoma-directed therapy may be indicated given the critical nature of the disease process.

Lymphoma-associated HLH is rare with case reports in the literature. Shah et al. reported a case of *ALK*-positive ALCL in a fifteen-year-old male with a similar acute presentation and rapid decline as seen in our patient [6]. Xu and Burns report a case of *ALK*-negative ALCL in an HIV-positive male who fulfilled 6 out of 8 diagnostic criteria for HLH and succumbed to the disease within two weeks [7]. On the other hand, Sovinz et al. report a case of a 15-year-old male treated for HLH with the HLH-94 protocol following which a diagnosis of *ALK*-negative ALCL was made. He was then treated with ALCL 99 International Protocol, remaining in remission at four years of follow-up [8].

As described in our case and reported cases in the literature, the importance of early recognition and treatment for HLH cannot be emphasized enough. Even though treatment of the primary disorder in secondary HLH is the treatment of choice, lack of a definitive primary diagnosis must not delay HLH-directed treatment. The possibility of an underlying disease must be considered and appropriate biopsy specimens be obtained towards the goal.

## 4. Conclusion

Coexistent DIC is uncommon and can delay a diagnosis of HLH. Our patient was started on HLH-directed therapy based on clinical judgement, and later, she fulfilled five diagnostic criteria. Hence, prompt recognition and treatment of the underlying disorder is crucial to improve the outcomes.

## Figures and Tables

**Table 1:** Diagnostic workup.

Laboratory parameter (units)	Normal range	Initial workup	Day 7	Day 20
Hemoglobin (g/dL)	14.0–18.0	13	8.5	8.7
White blood cells (10^9^/L)	4.8–10.8	2.61	2.99	0.37
Platelets (10^9^/L)	130–400	92	61	77
PT (seconds)	9.95–12.87	12.5	13.7	12.5
PTT (seconds)	27.0–39.2	28.7	99	28.7
Lactate dehydrogenase (IU/L)	60–200	—	982	674
Fibrinogen (mg/dL)	200–570	—	84	650
Haptoglobin (mg/dL)	34–200	—	<20	<20
Ferritin (ng/mL)	15–150	—	32,522	19,947
Total bilirubin (mg/dL)	0.2–1.2	1.0	1.3	1.0
Direct bilirubin (mg/dL)	0.0–0.2	0.46	0.49	0.35
Alkaline phosphatase (IU/L)	30–115	212	274	210
Aspartate aminotransferase (IU/L)	0–41	166	122	25
Alanine aminotransferase (IU/L)	0–45	61	37	49
